# Supplementary material for: Mutations in PCYT2 disrupt etherlipid biosynthesis and cause a complex hereditary spastic paraplegia
Source: Brain. 2019 Oct 22;142(11):3382–97. doi: 10.1093/brain/awz291 (PMC6821184; doi:10.1093/brain/awz291)
Supplement: awz291_Supplementary_Data [file awz291_supplementary_data.zip › awz291-Suppl_data/Supplementary_Data4.zip!/Supplementary information 3 - fibroblast lipidomics.zip!/Fibroblast lipidomics/quality.pdf]

CORE FACILITY METABOLOMICS
Laboratory Genetic Metabolic Diseases
Meibergdreef 9
1105 AZ Amsterdam

Frédéric Vaz +31 20 5662890
Michel van Weeghel +31 20 5661918

October 29, 2018

QUALITY CONTROL REPORT

Quality control analysis performed for:
Michel van Weeghel

Project manager and contact AMC:
Michel van Weeghel
m.vanweeghel@amc.nl

Quality control analysis was performed by Martin Vervaart
Bioinformatics analysis was performed by Angela Luyf, Michel van Weeghel

Report version 2018.002

Quality control of the lipidomics data

In this section the quality of the lipidomics data is analysed and explained.

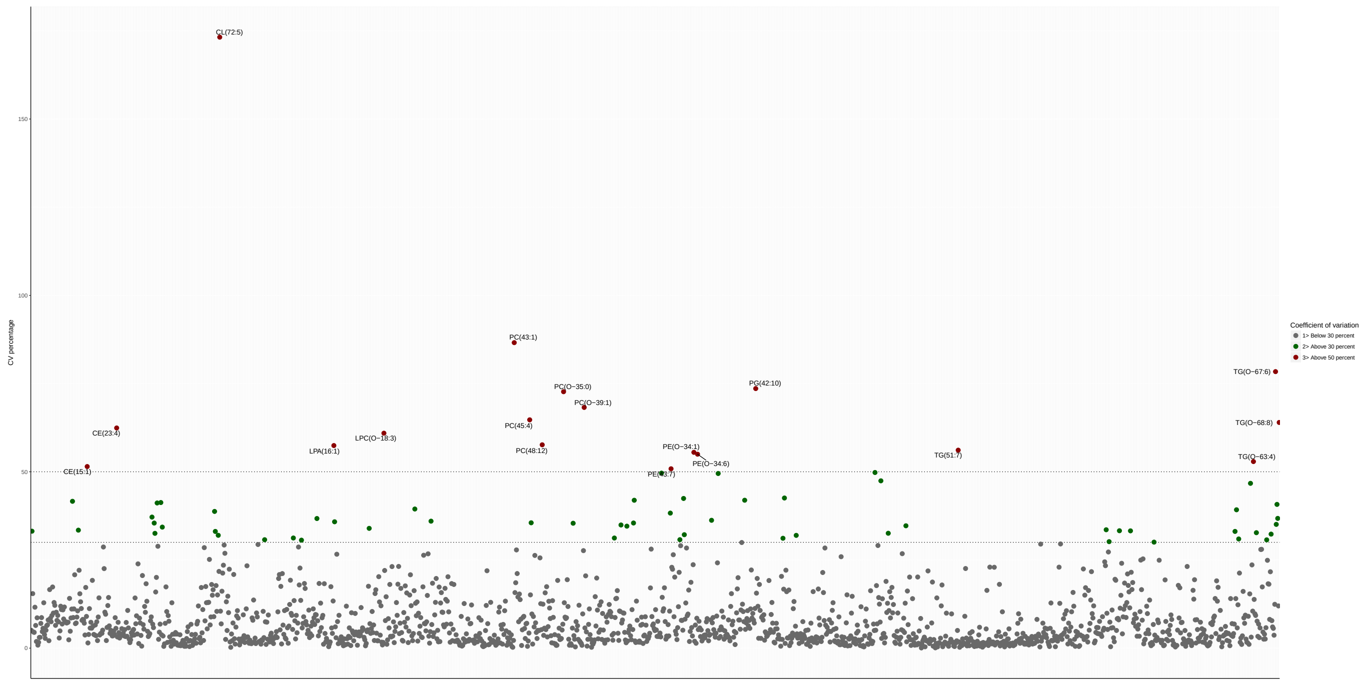

Figure 1: Quality control plot showing the Coefficient of Variance in percentage. The cuttoff is set on 50%

Quality control is a pool of all samples analyzed. This sample is injected after every 10 injections. The data represents the Coefficient of Variance in percentage for each metabolite (Figure 1). The pie chart (Figure 2) indicates the actual quality of the lipidomics data. Good quality data is the lipidomics data with a %CV of less than 30. The moderate lipidomics data is the data with a %CV between 30 and 50. The use with caution data is lipidomics data with a %CV greater than 50. The lipid species which have a %CV greater than 50 are also shown in Table 3. Interpretation of these lipid species should be done with caution as Coefficient of Variance in this data is large.

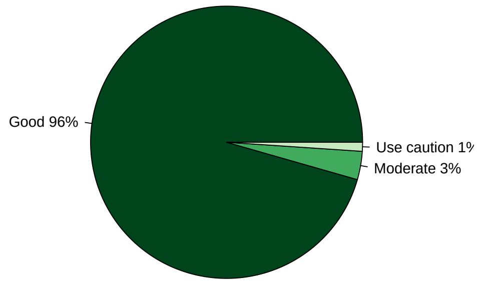

Figure 2: Pie chart indicating the quality of the lipidomics data

Metabolite	Average	SD	CV
CE(15:1)	4.4624	2.2973	51.4813
CE(23:4)	18.3555	11.4574	62.4195
CL(72:5)	5.9606	10.3241	173.2051
LPA(16:1)	1.2247	0.7034	57.4338
LPC(O-18:3)	0.2960	0.1804	60.9384
PC(43:1)	0.1654	0.1432	86.6092
PC(45:4)	0.2216	0.1435	64.7298
PC(48:12)	0.0918	0.0529	57.6495
PC(O-35:0)	0.4077	0.2964	72.6995
PC(O-39:1)	0.2291	0.1563	68.2420
PE(43:7)	0.4579	0.2328	50.8512
PE(O-34:1)	27.9352	15.5074	55.5122
PE(O-34:6)	0.3344	0.1839	54.9797
PG(42:10)	0.4299	0.3163	73.5876
TG(51:7)	0.0824	0.0462	56.1279
TG(O-63:4)	0.0101	0.0053	52.8969
TG(O-67:6)	0.0036	0.0028	78.3937
TG(O-68:8)	0.0029	0.0018	63.9821

Table 2: Lipids with a %CV higher than 50 (be carefull with the interpretation using these lipids)
